# Effects of climate and plant functional types on forest above-ground biomass accumulation

**DOI:** 10.1186/s13021-023-00225-1

**Published:** 2023-03-22

**Authors:** Xia Chen, Mingyu Luo, Markku Larjavaara

**Affiliations:** 1 Institute of Ecology and Key Laboratory for Earth Surface Processes of the Ministry of Education, College of Urban and Environmental Sciences, Peking University, Beijing, China; 2 Department of Forest Sciences, University of Helsinki, Helsinki, Finland

**Keywords:** Above-ground biomass, Bayesian statistics, Climate, Forest functional types, Growth model, Stand age

## Abstract

**Background:**

Forest above-ground biomass (AGB) accumulation is widely considered an important tool for mitigating climate change. However, the general pattern of forest AGB accumulation associated with age and climate gradients across various forest functional types at a global scale have remained unclear. In this study, we compiled a global AGB data set and applied a Bayesian statistical model to reveal the age-related dynamics of forest AGB accumulation, and to quantify the effects of mean annual temperature and annual precipitation on the initial AGB accumulation rate and on the saturated AGB characterizing the limit to AGB accumulation.

**Results:**

The results of the study suggest that mean annual temperature has a significant positive effect on the initial AGB accumulation rate in needleleaf evergreen forest, and a negative effect in broadleaf deciduous forest; whereas annual precipitation has a positive effect in broadleaf deciduous forest, and negative effect in broadleaf evergreen forest. The positive effect of mean annual temperature on the saturated AGB in broadleaf evergreen forest is greater than in broadleaf deciduous forest; annual precipitation has a greater negative effect on the saturated AGB in deciduous forests than in evergreen forests. Additionally, the difference of AGB accumulation rate across four forest functional types is closely correlated with the forest development stage at a given climate.

**Conclusions:**

The contrasting responses of AGB accumulation rate to mean annual temperature and precipitation across four forest functional types emphasizes the importance of incorporating the complexity of forest types into the models which are used in planning climate change mitigation. This study also highlights the high potential for further AGB growth in existing evergreen forests.

**Supplementary Information:**

The online version contains supplementary material available at 10.1186/s13021-023-00225-1.

## Background

Curbing dangerous climate change requires both drastic emissions cuts and the removal of large carbon dioxide quantities from the atmosphere [1]. Forest above-ground biomass (AGB), referring to the accumulated dry matter resulting from photosynthesis and tree growth, is an important carbon stock because nearly 50% of AGB is carbon [2]. The role that forests play in mitigating global warming depends on the rate at which carbon is assimilated by trees and converted into biomass, along with the quantity and persistence of this biomass in the forests [3, 4]. Positively influencing forest area change, including decelerating deforestation [5–7] or accelerating reforestation [1, 3, 8] has widely been discussed as a potential large-scale method for curbing climate change. However, the levels of C storage vary with forest development stages, the increasing the C density of successional forests may be an equally important method for mitigating climate change.

Biomass accumulation during forest stand development is one of the most paradigmatic processes in ecology [9]. Generally, the feedback between forest structure which influences light environments and tree growth may control forest dynamics [10]. The causes behind age- or size-related declines in biomass accumulation have been and remain widely discussed. The increasing respirational cost of larger AGB causes the productivity decline [11], and the reorganization of the forest canopy and changes in population structure following canopy closure lead directly to the decline in stand growth [12]. Also, the age-related decline of stand biomass accumulation could be attributable to declined production, increased mortality or both [13].

The response of forests to environmental variations has spatial variability [14]. Several studies have shown that mean annual temperature is an important determinant of the spatial distribution of biomass, due to its effect on the ecophysiological processes that control the net primary productivity rate [15]. Temperature variables, the mean annual temperature or temperature seasonality, could explain 19–71% of the variation in the C fluxes analysis [16]. Meanwhile, the effects of temperature on C storage might be moderated by moisture availability and water pressure deficit [17]. Because precipitation could influence water availability, which in turn affects stomatal conductance, nutrient uptake, leaf area index, and thus tree’s productivity across a broad range of forest types [18]. Also, the interactions between temperature and precipitation may influence C fluxes [19]. The responses of forest biomass accumulation to the climate largely depend on plant functional types (PFTs) [20, 21]. The contrasting responses of growth to temperature in angiosperm and coniferous trees have been demonstrated in several studies. For example, increased temperatures had positive effects on the tree growth of deciduous broadleaved species, but neutral or negative effects on conifers in Mediterranean forests [22, 23]. Deciduous broadleaved species show stronger growth (measured as stem diameter and biomass) responses to elevated temperature when compared with evergreen trees and conifers [24]. By contrast, the effect of precipitation on growth was much more uniform across tree species than that of temperature. A reduction in precipitation was predicted to decrease tree growth in angiosperm and coniferous trees [23]. However, a recent study reported that low precipitation favors the growth of deciduous broad-leaved trees [25]. An in-depth understanding of functional type-specific forest growth responses to climate and age variability is necessary for evaluating the potential of forest AGB accumulation in global climate change mitigation [26]. Forest growth responses to climate warming still include many uncertainties, especially at the functional type level [27]. The global patterns of these contrasting growth and distributional responses among divergent tree functional types to climate still need to be further studied.

Understanding plant functional-related variations in biomass accumulation is critical for reducing uncertainty regarding the potential for carbon uptake and climate change mitigation through forest regrowth. The largest contributor to the increment of vegetation C storage between 2010 and 2050 in China’s forests was deciduous broadleaf forest, followed by evergreen needleleaf forest, while the smallest was deciduous needleleaf forest. The vegetation C sequestration rate per area also varied significantly between different forest types, deciduous broadleaf forest had the highest C sequestration rate, while deciduous needleleaf forest had the lowest [28]. The resource (light and water) use efficiency of evergreen forests increased initially and then gradually declined after reaching the mature stage, while deciduous forest resource use efficiency continuously increased with age [29].

The complexity of forest types has been increasingly noted in the AGB estimation modeling [30], however, more up-to-date information concerning the dynamics and spatial patterns of AGB accumulation across diverse forest functional types with stand development is necessary. Therefore, in this study, we applied a Bayesian statistical model to fit the relationship between AGB and age in various forest functional types based on leaf morphology (broadleaf vs. needleleaf) and leaf phenology (deciduous vs. evergreen).

## Methods

### Overview

In this research, we developed a model of global forest AGB as a function of age, mean annual precipitation (MAP) and mean annual temperature (MAT). We then fitted the parameters of the model with a Bayesian method using a global AGB data set that we compiled. Based on the parameterized model and the global forest age data set, we then predicted global forest current AGB, saturated AGB, and the AGB accumulation potential (Additional file 2: Fig. S1).

### Statistical model of forest AGB accumulation

We conducted separate analyses for four forest functional types (needleleaf evergreen, needleleaf deciduous, broadleaf evergreen, and broadleaf deciduous). For each forest functional type, suppose that we have $n$ data points, labeled $i=\mathrm{1,2},\dots ,n$. Let ${\mathrm{AGB}}_{i}$ be the observed value of AGB. It is modeled as

1 $${\mathrm{AGB}}_{i} \sim \mathrm{Normal }\left({y}_{i},{\left({y}_{i}\sigma \right)}^{2}\right)$$

where ${y}_{i}$ denotes the expected value of the observed AGB. The standard deviation is modeled as ${y}_{i}\sigma$, where $\sigma$ is the proportion of standard deviation contributed by a unit of mean AGB value. The mean value ${y}_{i}$ is modeled as a Monod function of age [31, 32], i.e.,

2 $${y}_{i}={\mu }_{i}\frac{{a}_{i}}{{a}_{i}+{\mu }_{i}/{r}_{i}}$$

where ${r}_{i}$ is the initial AGB accumulation rate,${a}_{i}$ is forest age, and ${\mu }_{i}$ is the saturated AGB (Mg ha^–1^). The realized AGB accumulation rate (Mg ha^–1^ year^−1^) is the derivative of $y$ respective to $a$, that is

3 $$\frac{\partial y}{\partial a}=r\frac{1}{{\left(1+\frac{\mu }{r}a\right)}^{2}}$$

In the initial stage of forest growth, $a=0$, the accumulation rate is equal to $r$. As forest age increases, the accumulation rate will decrease.

We assume that ${r}_{i}$ and ${\mu }_{i}$ have a linear dependence on MAP and MAT

4 $${\mu }_{i}={\beta }_{\mu ,0}+{\beta }_{\mu ,1}{z}_{i,1}+{\beta }_{\mu ,2}{z}_{i,2}$$

5 $${r}_{i}={\beta }_{r,0}+{\beta }_{r,1}{z}_{i,1}+{\beta }_{r,2}{z}_{i,2}$$

The covariates ${z}_{i,1}$ and ${z}_{i,2}$ are normalized MAP (mm) and MAT (℃). They are calculated as

6 $${z}_{i,1}=\frac{{\mathrm{MAP}}_{i }-800}{500}$$

and

7 $${z}_{i,2}=\frac{{\mathrm{MAT}}_{i }-7}{7}$$

to rescale them into variables with mean values near 0 and standard deviations near 1. This normalization will not change statistical and prediction results but will only improve computational efficiency by letting the slopes be at similar scales. We rescale the precipitation and temperature conditions for four forest types together (MAP = 800 mm and MAT = 7 ℃, which are mean values calculated from the climate data in the AGB data set compiled for fitting models), to directly compare their intercept terms under the same climate condition.

### AGB and climate data

**Fig. 1:**
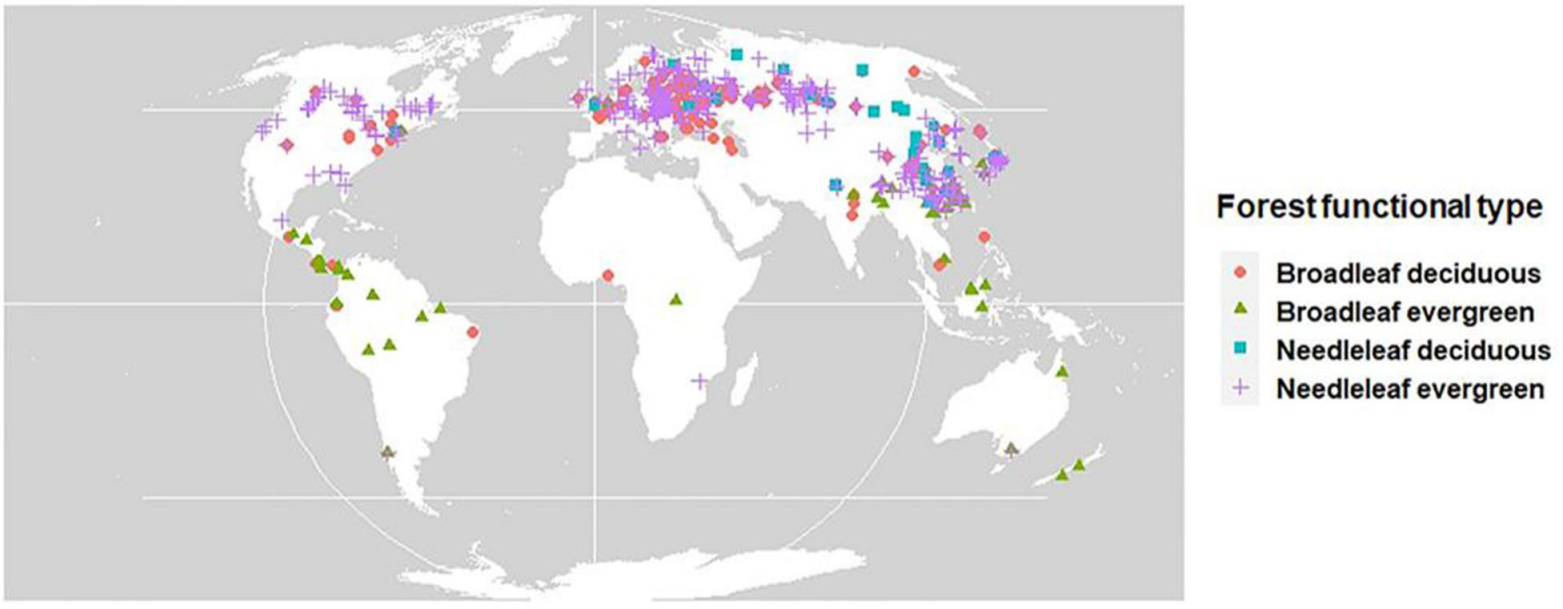
The studied sites at a global scale used as initial input data

To fit the parameters in our forest biomass accumulation model, we compiled a data set containing AGB, age, forest functional type, and geographical location. The data were collected in two ways. First, we searched available databases, including (a) the Global Forest Carbon Database [33, 34], (b) the Forest Biomass database of Eurasia [35], and (c) the Forest Biomass database of China [36]. Second, we conducted additional targeted literature searches to identify further available data on the AGB analyzed here. Details of our data collection can be found in “Data collection” in the Supporting Information (Additional file 1). Overall, the database consists of four forest functional types (Fig. 1): needleleaf evergreen (3839 records), needleleaf deciduous (322 records), broadleaf evergreen (596 records), and broadleaf deciduous (1623 records).

We extracted the monthly temperatures (near-surface air temperature) and precipitation (1970–2000) according to their geographic coordinates from WorldClim version 2.1 [37].

### Model fitting and evaluation

For each forest type, we used Bayesian methods [38] to obtain the posterior distribution of parameters ${\sigma }^{2}$,${\beta }_{\mu ,0}$, ${\beta }_{\mu ,1}$, ${\beta }_{\mu ,2}$,${\beta }_{r,0}$, ${\beta }_{r,1}$, and ${\beta }_{r,2}$. The posterior distributions were simulated using Markov chain Monte Carlo (MCMC) using R package rstan [39]. We performed 20 000 iterations for each chain (a total of four chains). The prior distributions are chosen as ${\sigma }^{2}\sim \mathrm{inv}\_\mathrm{Gamma}\left(\mathrm{1,1}\right)$, ${\beta }_{\mu ,0}\sim N\left({\mathrm{200,10}}^{6}\right)$, ${\beta }_{r,0}\sim N\left({\mathrm{2,10}}^{6}\right)$, and ${\beta }_{\mu ,1}$, ${\beta }_{\mu ,2}$, ${\beta }_{r,1}$, ${\beta }_{r,2}\sim N\left({\mathrm{0,10}}^{6}\right)$.

With a new data point $l$ for a given forest type, from the posterior sample of the parameters, we can obtain the posterior prediction sample of

8 $${y}_{l}={\mu }_{l}\frac{{a}_{l}}{{a}_{l}+{\mu }_{l}/{r}_{l}}$$

where ${r}_{l}$ and ${\mu }_{l}$ are calculated from Eq. 3 and Eq. 4 and are constrained in $[0,+\infty )$ (the negative values are set to 0). The predicted AGB value is calculated by

9 $${y}_{l,\mathrm{pred}}=\mathrm{median}\left({y}_{l}\right)$$

where ${y}_{l,\mathrm{pred}}$ is the posterior median of ${y}_{l}$.

To assess the model performance, we computed the coefficient of determination (${R}^{2}$) (Eq. 10) and the root-mean-square error (*RMSE*) (Eq. 11). The percentage root mean-square error (*RMSE%*) (Eq. 12) were used to evaluate the prediction performance of the models:

10 $${R}^{2}=1-\frac{\sum_{l=1}^{n}{\left({y}_{l}-{y}_{l,pred}\right)}^{2}}{\sum_{l=1}^{n}{\left({y}_{l}-\overline{y }\right)}^{2}}$$

11 $$RMSE=\sqrt{\sum_{l=1}^{n}\frac{{({y}_{l}-{y}_{l,\mathrm{pred}})}^{2}}{n}}$$

12 $$RMSE\%=\frac{RMSE}{\overline{y} }\times 100$$

where ${y}_{l}$ is the observed AGB value, ${y}_{l,\mathrm{pred}}$ is the predicted AGB value based on the model, $\overline{y }$ is the arithmetic mean of all the observed AGB values, and n is the sample number. In general, a higher ${R}^{2}$ value and lower RMSE and RMSE% values indicate a relatively better estimation performance of the model. In addition, we compare the observed values ${\mathrm{AGB}}_{l}$ and predicted values ${y}_{l,\mathrm{pred}}$ to evaluate our model. The residual is defined as ${y}_{l,\mathrm{pred}}-{y}_{l,\mathrm{obs}}$, and the residual fraction is defined as $\left({y}_{l,\mathrm{pred}}-{y}_{l,\mathrm{obs}}\right)/{y}_{l,\mathrm{pred}}$. We also conducted cross validation to evaluate our model fitting. For the data set of each forest type, we randomly chose 70% of the data points as a training set, to fit the model parameters, and let the other data points be the testing set, to evaluate the obtained predictions. To test the sensitivity of our analysis, we additionally used the climate data for the intervals 1961–1969 and 2001–2009 to fit the model. To quantify the spatial similarity between our model-predicted AGB and remote sensing-based AGB estimates, we resampled the 1-km resolution AGB (Mg ha^−1^) to a 0.5-degree grid from a wall-to-wall global forest AGB map [40], after which we extracted AGB to compare with the model-predicted AGB.

### Global forest AGB prediction

The Global Forest Age Dataset (GFAD) [41] includes both natural and managed forests. This data set was inferred based on existing forest attributes and combined with MODIS fire information, which allows the calculation of the combined effect of artificial or natural events that result in forest regrowth [42]. GFAD describes the forest age distributions of four plant functional types (needleleaf evergreen, needleleaf deciduous, broadleaf evergreen, and broadleaf deciduous) in a 0.5-degree grid (Additional file 2: Fig. S2). The GFAD data set represents the 2000–2010 period. Each grid cell contains 15 forest age classes (mapped from the MODIS Collection 5.1 land cover data set) of each forest functional type. The 15 forest age classes contain the forest areal fractions in the age intervals$\left[\mathrm{0,10}\right],\left[\mathrm{10,20}\right],\left[\mathrm{20,30}\right],\dots ,[\mathrm{130,140}]$, and$[140,+\infty )$. We use 5, 15, …,135, and 145 to represent forest age in each age class. The current forest AGB for each forest functional type in each age class is predicted by Eq. 7 and Eq. 8 and are multiplied by the areal fractions. The forest total AGB in each grid cell is calculated by summing the product of the AGB and the corresponding areal fraction of each forest functional type. Let ${w}_{i,j,k}$ be the areal fraction of forest type$j$, age class $k$ at location$i$, and let ${y}_{l,j,k,\mathrm{pred}}$ be the predicted AGB, and then the predicted value of the forest AGB, including all four forest functional types, is calculated as

13 $${Y}_{l,\mathrm{pred}}=\sum_{j=1}^{4}\sum_{k=1}^{15}{w}_{i,j,k}{y}_{l,j,k,\mathrm{pred}}$$

## Results

### Variations of above-ground biomass accumulation during forest stand development across forest functional types

**Fig. 2:**
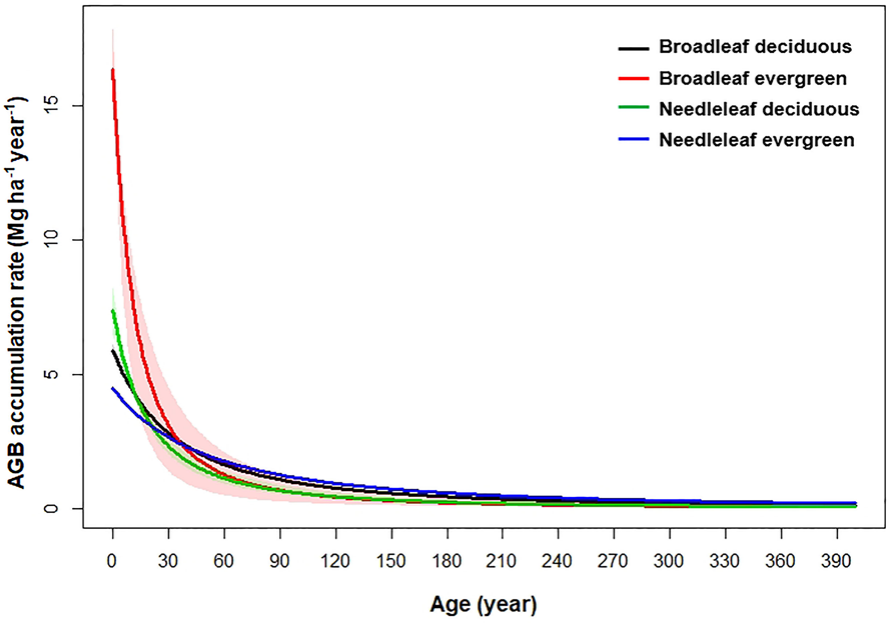
Posterior medians and 80% credible intervals of forest AGB accumulation rates (Mg ha^–1^ year.^−1^) with stand age across forest type, calculated using the intercept terms of maximum biomass and initial accumulation rates (annual precipitation = 800 mm and mean annual temperature = 7 ℃)

The AGB accumulation rate exhibited significant differences with respect to forest functional types. As shown in Fig. 2, the broadleaf evergreen forest had the highest initial AGB accumulation rate (*r* = 16.37 Mg ha^–1^ year^−1^), followed by needleleaf deciduous (*r* = 7.34 Mg ha^–1^ year^−1^), broadleaf deciduous (*r* = 5.89 Mg ha^–1^ year^−1^), and needleleaf evergreen forest (*r* = 4.46 Mg ha^–1^ year^−1^) (annual precipitation = 800 mm and mean annual temperature = 7 ℃). After the age of 25 years, the differences in accumulation rates between the functional types become relatively unimportant (Fig. 2). In addition, the AGB accumulation rate of the broadleaf evergreen forest is distinctly higher than that of the broadleaf deciduous forest when the age was less than 35 years, but the broadleaf deciduous forest had a higher AGB accumulation rate when exceeding 35 years. By contrast, in the needleleaf forests, the AGB accumulation rate of the deciduous forest was higher than that of the evergreen forest when the age was less than 25 years, after which the evergreen forest had a higher AGB accumulation rate (Fig. 2).

**Fig. 3:**
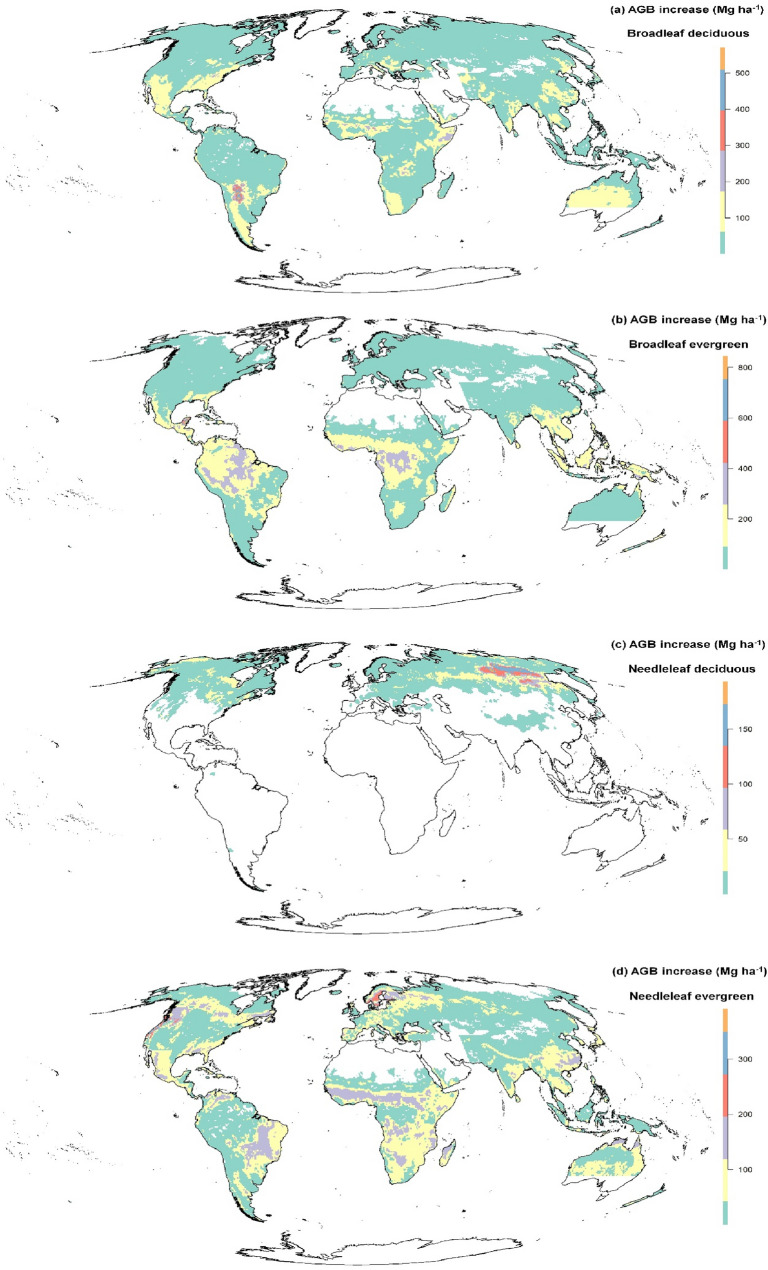
Posterior medians of AGB (Mg ha^−1^) accumulation potential (the difference between current and saturated AGB) at a resolution of a 0.5-degree of four forest types

Based on the parameter estimates and the global forest age data set, we quantified the potential of AGB accumulation of four forest functional types respectively with the median value of the posterior distribution at a resolution of a 0.5-degree (Fig. 3). The broadleaf evergreen forest had the highest mean AGB increase potential (50.83 ± 76.02 Mg ha^−1^) based on the difference between current and saturated AGB. As shown in Fig. 3b, broadleaf evergreen trees distributed in northern South America and central Africa had higher AGB increase potential. The mean AGB increase potential of the needleleaf evergreen forest was 42.9 ± 46.76 Mg ha^−1^, and the AGB accumulation potential of needleleaf evergreen trees in northwestern Eurasia and western North America was high (Fig. 3d). The mean AGB increase potential of the broadleaf deciduous forest was 29.17 ± 43.25 Mg ha^−1^, high AGB accumulation potential was occurred in south-central South America (Fig. 3a). The needleleaf deciduous forest had the lowest mean AGB increase potential (16.78 ± 26.72 Mg ha^−1^). Needleleaf deciduous trees distributed in northeast Eurasia had a higher AGB accumulation potential (Fig. 3c).

### Effects of climate on AGB accumulation across different forest functional types

In this study, we used estimated model parameters to quantify the effects of annual precipitation (MAP) and mean annual temperature (MAT) on the initial AGB accumulation rate and saturated AGB. Table 1 shows parameter estimates from the current model. The MAT effects quantify the *µ* and initial AGB accumulation rat changes per a 1 °C change in MAT, and the MAP effects quantify the saturated AGB and initial AGB accumulation rate changes per a 1 mm change in MAP for each forest type. Note that in our statistical model, MAP and MAT values are normalized, but the effects of MAP and MAT are reported in their original scales in Table 1. We consider the slope term statistically significant if its 95% credible interval does not contain 0. The initial AGB accumulation rate changes were closely correlated with climatic factors across the four functional types (Table 1). Specifically, MAT had a significant positive effect on the initial AGB accumulation rate of needleleaf evergreen forest but a significant negative effect on the initial AGB accumulation rate of broadleaf deciduous forest. By contrast, MAT had weak relationships with the initial AGB accumulation rate of needleleaf deciduous and broadleaf evergreen forests. The initial AGB accumulation rate of the broadleaf deciduous forest exhibited a positive correlation with MAP but showed a negative correlation with MAP in the broadleaf evergreen forest. In addition, the saturated biomass changes were also associated with climatic factors in all forest functional types (Table 1). MAT positively affected the saturated AGB of broadleaf deciduous, broadleaf evergreen, and needleleaf evergreen forests. Except for a positive effect of MAP on the saturated AGB of needleleaf evergreen forest, precipitation negatively affected the saturated AGB of the other three forest functional types. On a whole, the positive effect of MAT on the saturated AGB of the broadleaf evergreen forest was greater than on the broadleaf deciduous forest. Compared with evergreen forests, MAP had a greater negative effect on saturated AGB in deciduous forests.

**Table 1:** Posterior mean and 95% credible interval of the parameter estimates from the current model

Parameters	Broadleaf deciduous (n = 1623)	Broadleaf evergreen (n = 596)	Needleleaf deciduous (n = 322)	Needleleaf evergreen (n = 3839)
Intercept (${\beta }_{r,0}$) (Mg ha^–1^ year^−1^)	5.89 (5.55, 6.24)	16.37 (14.17, 18.77)	7.34 (5.85, 8.7)	4.46 (4.31, 4.61)
MAP effect ${(\beta }_{r,1})$ (Mg ha^–1^ year^−1^ mm^–1^)	**0.0071** **(0.0062, 0.0079)**	**−0.0032** **(−0.0037, −0.0027)**	0.0028 (−0.0015, 0.0080)	−0.0002 (−0.0005, 0.0001)
MAT effect ${(\beta }_{r,2})$ (Mg ha^–1^ year^−1^ °C^–1^)	**-0.0386** **(-0.0729, -0.0043)**	−0.1429 (−0.2871, 0.0114)	0.2486 (−0.0243, 0.4443)	**0.2686** **(0.2514, 0.2857)**
Intercept (${\beta }_{\mu ,0}$) (Mg ha^–1^)	393.55 (360.95, 430.72)	373.10 (182.5, 645.17)	286.30 (227.29, 369.82)	449.56 (416.6, 486.7)
MAP effect ${(\beta }_{\mu ,1})$ (Mg ha^–1^ mm^–1^)	**−0.2122** **(−0.2693, −0.1418)**	**−0.1905** **(−0.2621, −0.1210)**	**−0.1964** **(−0.3613, −0.0263)**	**0.0874** **(0.0064, 0.1745)**
MAT effect ${(\beta }_{\mu ,2})$ (Mg ha^–1^ °C^–1^)	**19.0471** **(15.3600, 22.6571)**	**36.9300** **(5.4271, 69.0714)**	8.2000 (−4.3529, 19.4900)	**22.3700** **(18.7143, 26.0643)**
Variance of error ($\sigma$)	0.52 (0.49,0.54)	0.44 (0.41,0.47)	0.5 (0.46,0.52)	0.52 (0.5,0.53)

All parameters have credibility intervals non-overlapping with zero (bold type means statistically significant). Each forest type is fitted to the model separately. *r* is the initial biomass accumulation rate, and *µ* is the saturated AGB. MAP is mean annual precipitation and MAT is mean annual temperature

### Model evaluation and sensitivity analysis

**Fig. 4:**
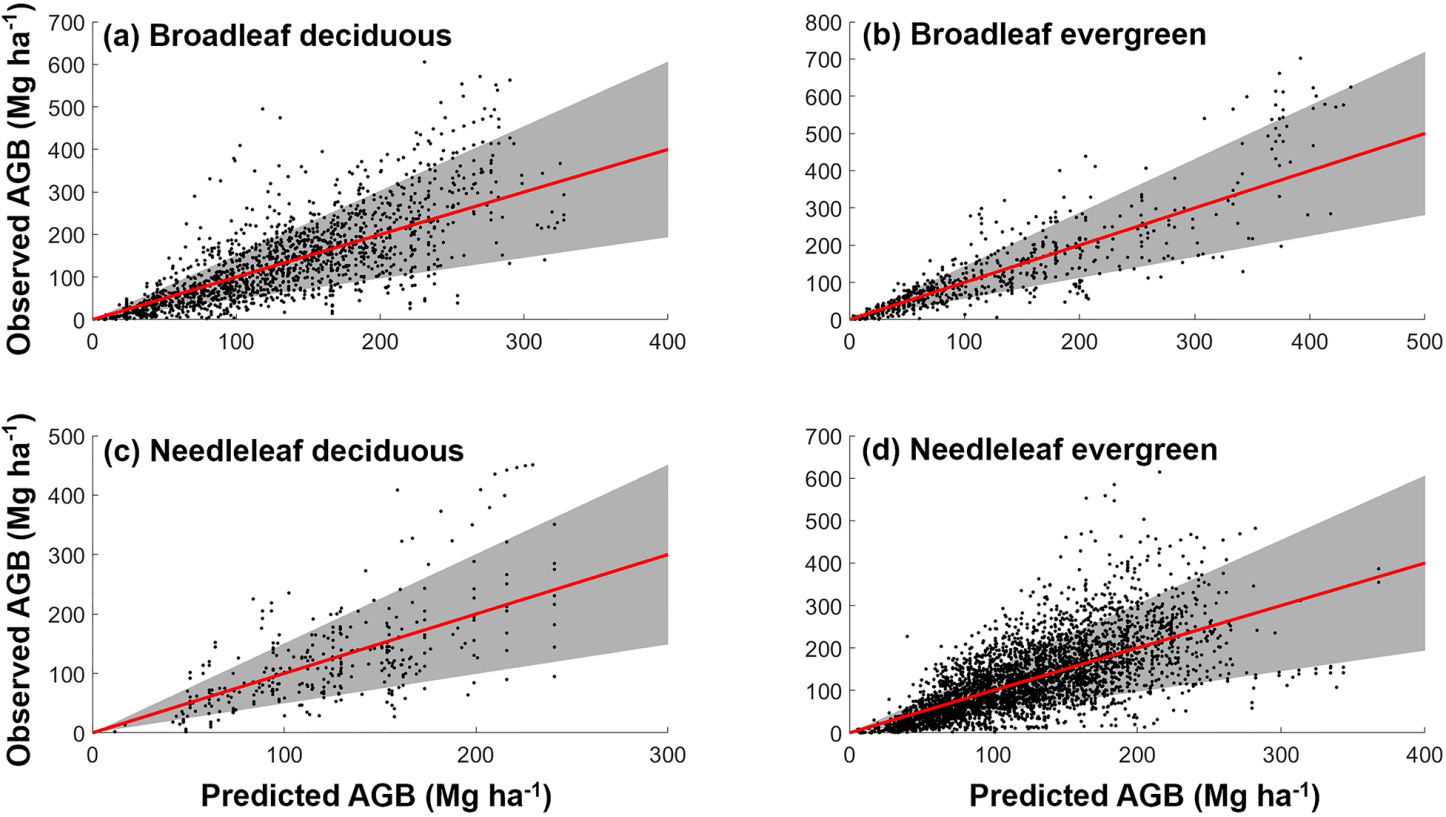
Model-predicted above-ground biomass (AGB) (Mg ha^−1^) plotted against observed AGB across four forest types: **a** Broadleaf deciduous, **b** Broadleaf evergreen, **c** Needleleaf deciduous, and **d** Needleleaf evergreen. The shaded area represents one standard deviation of the mean predicted AGB

As the deviation between the modeled AGB and the observations is increased with the scales of AGB values, we added heteroscedasticity terms to the model (Eq. 1). We plotted the observed AGB (Mg ha^−1^) against model-predicted AGB (Mg ha^−1^) in Fig. 4. The model of needleleaf deciduous and evergreen forests had the lower* R*^2^ value (0.40 and 0.46, respectively) with an *RMSE*% of 51.64% and 51.92%, respectively (Table 2). Conversely, the models of broadleaf deciduous and evergreen forests performed relatively better. The model of broadleaf forests had a higher *R*^2^ value (0.56 and 0.72, respectively) with an *RMSE*% of 49.35% and 49.27%, respectively (Table 2). The residual fractions (the ratio between residual and predicted value) of broadleaf forests were lower than needleleaf forests (Additional file 2: Figs. S3 and S4). Intuitively, the model of these four forest types was better at estimating AGB less than 400 Mg ha^−1^. We conducted a sensitivity analysis to evaluate the impacts of climate conditions on the estimated parameters for AGB prediction. The 95% credible intervals of the parameter estimate from the climate periods (1970–2000) in the current model overlapped with the 95% credible intervals of the parameter estimates from the other two different climate periods (1961–1969 and 2001–2009) (Additional file 2: Table S1).

**Table 2:** Summary of AGB estimation of the models for different forest functional types

Forest type	*R*^2^	*RMSE* (Mg ha^−1^)	*RMSE*%	Residual fraction (%)	Standard deviation (σ) (Mg ha^−1^)
Broadleaf deciduous	0.56	69.11	49.35	0.07	70.85
Broadleaf evergreen	0.72	67.61	49.27	0.21	61.80
Needleleaf deciduous	0.40	65.09	51.64	0.49	63.37
Needleleaf evergreen	0.46	64.97	51.92	52.16	63.32

## Discussion

### Forest AGB accumulation varied across functional types

Our results suggest that the effect of forest age on the AGB accumulation rate depended on forest functional type (Fig. 2). In a given climate (MAP = 800 mm and MAT = 7 ℃, which are mean values calculated from the climate data in the AGB data set compiled for fitting models), the broadleaf evergreen forest had a higher AGB accumulation rate than the broadleaf deciduous forest when the forest age was below 35; but the AGB accumulation rate of broadleaf deciduous forest was higher when the forest age exceeding 35 years. There are several possible explanations for this result. Evergreen forest resource use efficiencies initially increased before reaching the mature stage and then gradually declined, in contrast with deciduous forests, which exhibited continuously increasing trends with age [29]. Additionally, the variation of soil nitrogen (N) availability along with forest age also exhibits difference between evergreen and deciduous forests [43, 44]. Compared with evergreen forests, deciduous forests usually have a higher N mineralization rate due to their high-quality litter [45]. Therefore, deciduous forests can delay their functional decline by increasing soil fertility over time [46, 47]. However, the needleleaf forests exhibited an opposite dynamic in the variation of AGB accumulation rate between its evergreen and deciduous groups compared with the broadleaf forests (Fig. 2). This may attribute to the differences in photosynthetic performance [48] and hydraulic capacity [49] between broadleaf and needleleaf forests. These results reflect the high variability of the AGB accumulation rates along with age across various forest functional types.

The broadleaf evergreen and needleleaf evergreen forests showed a relatively high AGB increase potential (Fig. 3), suggesting the increasing biomass in existing evergreen forests might continue to take up atmospheric CO_2_ over the upcoming decades. However, we should note that there was a large uncertainty in the parameters of the broadleaf evergreen forest (Fig. 2). The insufficient sample size might be a reason caused this uncertainty. Additionally, forest productivity is also influenced by soil properties [50], and the variation of soil properties in the tropical forest might be larger than in other forest types. It should be noted that, the statistical model applied in this study assumed that climatic conditions do not fluctuate with time, we focused on the forest AGB accumulation potential without taking future climate change into account in our current study.

### The divergent response of AGB accumulation to climate across four forest functional types

Temperature and soil water availability are important environmental factors that may influence forest growth and development [51, 52]. In our study, the estimated model parameters were used to quantify the effects of annual mean temperature and annual precipitation on the saturated AGB and initial AGB accumulation rate. We found that the changes in the saturated AGB and initial AGB accumulation rate were closely correlated with climatic factors across the four functional types (Table 1). A study conducted in the Iberian Peninsula reported contrasting effects of temperature in Mediterranean angiosperms and conifers, there was a positive effect of rising temperatures on angiosperm tree growth, but neutral or negative effects on coniferous trees [22, 23]. However, the response of tree growth to the climate becomes more varied when the leaf attributes of deciduous and evergreen trees are added into consideration (Table 1). Our results showed that the direction and intensity of the influence caused by climatic factors (temperature and precipitation) on the saturated AGB and initial AGB accumulation rate of deciduous and evergreen forests are very different. Deciduous forests have time limits for photosynthesis each year and C allocation compared with evergreen forests [53], and the Weng et al. (2017) noted that temperature drives the distribution of deciduous and evergreen forests by affecting the N mineralization rates [54]. Overall, these physiological and phenological differences may modulate the divergent response across various forest types to environmental changes [23, 55]. We also observed that the effects of climatic factors on the initial AGB accumulation rate and saturated AGB could be contrasting in the same forest type. For example, temperature negatively affected the initial AGB accumulation rate of the broadleaf deciduous forest but positively affected its saturated AGB. This implies that age-related biological changes should be considered when evaluating forest functional responses to climate change and managing forest productivity [29]. Moreover, we found that precipitation had a negative effect on the saturated AGB of three forest types except the needleleaf evergreen forest (Table 1). It suggests that the positive correlation between precipitation and biomass [56–58] may not be an absolute pattern. A 100-mm increase in mean annual precipitation causes a 1.52% decrease in larch AGB in China [59]. Additionally, forest height exhibited a hump-shaped curve along a gradient of water availability, which suggests that excessive water supply negatively affects canopy height, possibly resulting in lower forest biomass [60]. Also, the highest biomass C density in a temperate moist forest occurs in moderately wet conditions [61]. Furthermore, our results show that the effect of precipitation on the initial AGB accumulation rate is largely inconsistent with its effect on saturated AGB for these four forest functional types (Table 1), reflecting an age dependence of this influence. One possible explanation is related to how precipitation affects organ biomass allocation (above- and below- ground) in various age stages [62].

### Uncertainty analysis of biomass model predictions and improvement

Understanding the robustness of the models and reliability of estimates requires an evaluation of estimation results. With the median value of the posterior distribution, the total AGB in each grid cell is calculated based on the age and proportion of the four forest functional types at a 0.5-degree resolution. The coefficient of determination (*R*^2^) between the model-predicted total forest AGB and the global wall-to-wall remote sensing-based AGB [40] (Hu et al., 2016) was 0.69 (*P* < 0.001) (Additional file 2: Fig. S5). The plot location uncertainty brought unneglectable error in their remote sensing-based forest AGB estimation [40]. In forest succession studies, the approach assuming the variation in biomass over time could be approximated by the variation across space was common [32]. However, the space-for-time approach used in this study might introduce some uncertainties because we could not incorporate the soil nutrients and properties into the model. In our study, we computed the uncertainty caused by the posterior distribution of parameter estimates (upper and lower quartiles) (Additional file 2: Fig. S6). However, the difficulty in collecting sufficient observation data including four forest functional types was a constraint for evaluating biomass estimates, the spatial uncertainty analysis and error budget of the forest AGB map was not fully quantified in this study. Moreover, in the current work, we mainly focus on the effects of species characteristics and climate on above-ground biomass accumulation, however, the forest productivity could also be influenced by soil characteristics and other factors such as natural and human disturbances and forest management. This reminds us that more comprehensive data should be collected to further enrich the model structure and therefore enhance the validity of the estimates of the proposed model and its application.

## Conclusion

In this study, we applied a Bayesian statistical model to fit the relationship between AGB and age in different forest functional types based on leaf morphology and phenology. Our results clearly showed the dynamics and spatial patterns of AGB accumulation across diverse forest functional types with stand development, and the contrasting responses of AGB accumulation to temperature and precipitation across four forest functional types. These findings highlighted the importance of incorporating the complexity of forest types into the forest biomass modeling. Moreover, high AGB accumulation potential of the evergreen forest implies that the increasing biomass in existing evergreen forest could play a crucial role in carbon storage and global warming mitigation.

## Supplementary Information

**Additional file 1. **Data Collection.

**Additional file 2: Figure S1.** A flowchart of the study. The blue arrow indicates the main working path, the red arrow indicates data input, and the yellow arrow indicates model evaluation. **Figure S2.** Age distributions of four forest functional types ((a) Broadleaf deciduous, (b) Broadleaf evergreen, (c) Needleleaf deciduous and (d) Needleleaf evergreen) at 0.5-degree grid from the Global Forest Age Dataset (GFAD) (Poulter et al., 2019). **Figure S3.** Residual fraction of the model prediction against the predicted AGB (Mg ha^−1^) across four forest types. The residual is defined as the difference between observed and predicted AGB. The residual fraction is the ratio between residual and predicted AGB. The red lines indicate the zero residual and the data points above it represent cases where predicted AGB is smaller than observed AGB. **Figure S4.** Cross-validation results of model fitting. For the data set of each forest type, we randomly chose 70% data points as the training set, to fit the model parameters and let the other data points be used as the testing set to evaluate the predictions. **Figure S5.** The comparison between predicted AGB (Mg ha^−1^) calculated based on the fitted model and a global wall-to-wall remote sensing-based AGB (Mg ha^−1^) maps made by Hu et al. (2016). The 1-km resolution wall-to-wall global forest AGB map was resampled and extracted at a 0.5-degree resolution. The mean residual is $33.32$ Mg ha^−1^ and the mean residual fraction is 0.199. **Figure S6.** Global geographic distribution of the predicted current (2000–2010 era) forest AGB (Mg ha^−1^). The total AGB in each grid cell is calculated based on the age and proportion of the four forest functional types at a 0.5-degree resolution with the median value (a), lower quartiles (b) and upper quartiles (c) of the posterior distribution. The blank area in southern Australia occurs because no data for this area exist in the global forest age data set. Other blank areas show grid cells without any forests due to glaciers or extreme aridity. **Table S1.** Sensitivity analysis of climate conditions on parameter estimation.

## Data Availability

All data supporting the results are open access and have been cited in the paper. For the AGB data, The Global Forest Carbon database is available at forc-db.github.io/. The Forest Biomass database of Eurasia is available at https://doi.org/10.1594/PANGAEA.871492. The Forest Biomass database of China is available at https://doi.org/10.6084/m9.figshare.c.3306930.v1. AGB data from published literature collected by the author is available from the corresponding author on request. The global forest age data set (GFAD) is available at https://doi.org/10.1594/PANGAEA.897392

## References

[1] Bastin JF, Finegold Y, Garcia C, Mollicone D, Rezende M, Routh D, Zohner CM, Crowthe TW (2009). The global tree restoration potential. Science.

[2] Martin AR, Doraisami M, Thomas SC (2018). Global patterns in wood carbon concentration across the world’s trees and forests. Nat Geosci.

[3] Griscom BW, Adams J, Ellis PW, Houghton RA, Lomax G, Miteva DA (2017). Natural climate solutions. Proc Natl Acad Sci USA.

[4] Lewis SL, Wheeler CE, Mitchard ETA, Koch A (2019). Regenerate natural forests to store carbon. Nature.

[5] Grainger A, Boucher DH, Frumhoff PC, Laurance WF, Lovejoy T, McNeely J (2009). Biodiversity and REDD at Copenhagen. Curr Biol.

[6] Godoy FL, Tabor K, Burgess ND, Mbilinyi BP, Kashaigili JJ, Steininger MK (2011). Deforestation and CO_2_ emissions in coastal Tanzania from 1990 to 2007. Environ Conserv.

[7] Austin KG, Baker JS, Sohngen BL, Wade CM, Daigneault A, Ohrel SB (2020). The economic costs of planting, preserving, and managing the world's forests to mitigate climate change. Nat Commun.

[8] Hawes M (2018). Planting carbon storage. Nat Clim Chang.

[9] Wardle DA, Bardgett RD, Klironomos JN, Setala H, Van Der Putten WH, Wall DH (2004). Ecological linkages between aboveground and belowground biota. Science.

[10] Moorcroft PR, Hurtt GC, Pacala SW (2001). A method for scaling vegetation dynamics: the ecosystem demography model (ED). Ecol Monogr.

[11] Odum EP (1969). The strategy of ecosystem development: an understanding of ecological succession provides a basis for resolving man's conflict with nature. Science.

[12] Smith FW, Long JN (2001). Age-related decline in forest growth: an emergent property. For Ecol Manag.

[13] Xu CY, Turnbull MH, Tissue DT, Lewis JD, Carson R, Schuster WS, Whitehead D, Walcroft AS, Li J, Griffin KL (2012). Age-related decline of stand biomass accumulation is primarily due to mortality and not to reduction in NPP associated with individual tree physiology, tree growth or stand structure in a Quercus-dominated forest. J Appl Ecol.

[14] Fauset S, Gloor M, Fyllas NM, Phillips OL, Asner GP, Baker TR, Patrick Bentley L, Brienen RJ, Christoffersen BO, del Aguila-Pasquel J, Doughty CE (2019). Individual-based modeling of Amazon forests suggests that climate controls productivity while traits control demography. Front Earth Sci.

[15] Bennett AC, Penman TD, Arndt SK, Roxburgh SH, Bennett LT (2020). Climate more important than soils for predicting forest biomass at the continental scale. Ecography.

[16] Banbury Morgan R, Herrmann V, Kunert N, Bond-Lamberty B, Muller-Landau HC, Anderson-Teixeira KJ (2021). Global patterns of forest autotrophic carbon fluxes. Glob Chang Biol.

[17] Poorter L, Bongers F, Aide TM, Almeyda Zambrano AM, Balvanera P, Becknell JM, Boukili V (2016). Biomass resilience of Neotropical secondary forests. Nature.

[18] Sandel B, Goldstein LJ, Kraft NJ, Okie JG, Shuldman MI, Ackerly DD (2010). Contrasting trait responses in plant communities to experimental and geographic variation in precipitation. New Phytol.

[19] Yu G, Chen Z, Piao S, Peng C, Ciais P, Wang Q (2014). High carbon dioxide uptake by subtropical forest ecosystems in the East Asian monsoon region. PNAS.

[20] Lin D, Xia J, Wan S (2010). Climate warming and biomass accumulation of terrestrial plants: a meta-analysis. New Phytol.

[21] Coll M, Peñuelas J, Ninyerola M, Pons X, Carnicer J (2013). Multivariate effect gradients driving forest demographic responses in the Iberian Peninsula. For Ecol Manag.

[22] Gómez-Aparicio LO, García-Valdés RA, Ruíz-Benito PA, Zavala MA (2011). Disentangling the relative importance of climate, size and competition on tree growth in Iberian forests: implications for forest management under global change. Glob Chang Biol.

[23] Carnicer J, Barbeta A, Sperlich D, Coll M, Peñuelas J (2013). Contrasting trait syndromes in angiosperms and conifers are associated with different responses of tree growth to temperature on a large scale. FRONT PLANT SCI.

[24] Way DA, Oren R (2010). Differential responses to changes in growth temperature between trees from different functional groups and biomes: a review and synthesis of data. Tree Physiol.

[25] Jin Y, Qian H (2023). Drivers of the differentiation between broad-leaved trees and shrubs in the shift from evergreen to deciduous leaf habit in forests of eastern Asian subtropics. Plant Divers.

[26] Boucher-Lalonde V, Morin A, Currie DJ (2012). How are tree species distributed in climatic space? A simple and general pattern. Global Ecol Biogeogr.

[27] Augusto L, De Schrijver A, Vesterdal L, Smolander A, Prescott C, Ranger J (2015). Influences of evergreen gymnosperm and deciduous angiosperm tree species on the functioning of temperate and boreal forests. Biol Rev.

[28] He N, Wen D, Zhu J, Tang X, Xu L, Zhang L (2017). Vegetation carbon sequestration in Chinese forests from 2010 to 2050. Glob Chang Biol.

[29] Xu H, Xiao J, Zhang Z, Ollinger SV, Hollinger DY, Pan Y (2020). Canopy photosynthetic capacity drives contrasting age dynamics of resource use efficiencies between mature temperate evergreen and deciduous forests. Glob Chang Biol.

[30] Li Y, Li M, Liu Z, Li C (2020). Combining Kriging interpolation to improve the accuracy of forest aboveground biomass estimation using remote sensing data. IEEE Access.

[31] Monod J (1949). The growth of bacterial cultures. Annu Rev Microbiol.

[32] Zhu K, Zhang J, Niu S, Chu C, Luo Y (2018). Limits to growth of forest biomass carbon sink under climate change. Nat Commun.

[33] Anderson-Teixeira KJ, Wang MM, McGarvey JC, LeBauer DS (2016). Carbon dynamics of mature and regrowth tropical forests derived from a pantropical database (T rop F or C-db). Glob Chang Biol.

[34] Anderson-Teixeira KJ, Wang MM, McGarvey JC, Herrmann V, Tepley AJ, Bond-Lamberty B (2018). ForC: a global database of forest carbon stocks and fluxes. Ecology.

[35] Schepaschenko D, Shvidenko A, Usoltsev V, Lakyda P, Luo Y, Vasylyshyn R (2017). A dataset of forest biomass structure for Eurasia. Scientific data.

[36] Luo Y, Zhang X, Wang X, Lu F (2014). Biomass and its allocation of Chinese forest ecosystems: ecological Archives E095–177. Ecology.

[37] Fick SE, Hijmans RJ (2017). WorldClim 2: new 1-km spatial resolution climate surfaces for global land areas. Int J Climatol.

[38] van de Schoot R, Depaoli S, King R, Kramer B, Märtens K, Tadesse MG (2021). Bayesian statistics and modelling. Nat Rev Methods Primers.

[39] Carpenter B, Gelman A, Hoffman MD, Lee D, Goodrich B, Betancourt M (2017). Stan: a probabilistic programming language. J Stat Softw.

[40] Hu T, Su Y, Xue B, Liu J, Zhao X, Fang J (2016). Mapping global forest aboveground biomass with spaceborne LiDAR, optical imagery, and forest inventory data. Remote Sens.

[41] Poulter B, Aragão L, Andela N, Bellassen V, Ciais P, Kato T, et al. The global forest age dataset and its uncertainties (GFADv1. 1).

[42] Pugh TA, Lindeskog M, Smith B, Poulter B, Arneth A, Haverd V (2019). Role of forest regrowth in global carbon sink dynamics. PNAS.

[43] Mueller KE, Hobbie SE, Oleksyn J, Reich PB, Eissenstat DM (2012). Do evergreen and deciduous trees have different effects on net N mineralization in soil?. Ecology.

[44] Takashima T, Hikosaka K, Hirose T (2004). Photosynthesis or persistence: nitrogen allocation in leaves of evergreen and deciduous Quercus species. Plant Cell Environ.

[45] Cornwell WK, Cornelissen JH, Amatangelo K, Dorrepaal E, Eviner VT, Godoy O (2008). Plant species traits are the predominant control on litter decomposition rates within biomes worldwide. Ecol Lett.

[46] Forrester DI, Collopy JJ, Beadle CL, Baker TG (2013). Effect of thinning, pruning and nitrogen fertiliser application on light interception and light-use efficiency in a young Eucalyptus nitens plantation. For Ecol Manag.

[47] Vitousek P (1982). Nutrient cycling and nutrient use efficiency. Am Nat.

[48] Klein T, Ramon U (2019). Stomatal sensitivity to CO_2_ diverges between angiosperm and gymnosperm tree species. Funct Ecol.

[49] Lusk CH, Wright I, Reich PB (2003). Photosynthetic differences contribute to competitive advantage of evergreen angiosperm trees over evergreen conifers in productive habitats. New Phytol.

[50] Grigal DF, Vance ED (2000). Influence of soil organic matter on forest productivity. NZ J FORESTRY SCI.

[51] Pausas JG, Austin MP (2001). Patterns of plant species richness in relation to different environments: an appraisal. J Veg Sci.

[52] Saiter FZ, Eisenlohr PV, Barbosa MR, Thomas WW, Oliveira-Filho AT (2016). From evergreen to deciduous tropical forests: how energy–water balance, temperature, and space influence the tree species composition in a high diversity region. Plant Ecol Divers.

[53] Genet H, Bréda N, Dufrene E (2010). Age-related variation in carbon allocation at tree and stand scales in beech (*Fagus sylvatica* L.) and sessile oak (*Quercus petraea* (Matt.) Liebl.) using a chronosequence approach. Tree Physiol.

[54] Weng E, Farrior CE, Dybzinski R, Pacala SW (2017). Predicting vegetation type through physiological and environmental interactions with leaf traits: evergreen and deciduous forests in an earth system modeling framework. Glob Chang Biol.

[55] Meinzer FC, Woodruff DR, Eissenstat DM, Lin HS, Adams TS, McCulloh KA (2013). Above-and belowground controls on water use by trees of different wood types in an eastern US deciduous forest. Tree Physiol.

[56] Álvarez-Dávila E, Cayuela L, González-Caro S, Aldana AM, Stevenson PR, Phillips O (2017). Forest biomass density across large climate gradients in northern South America is related to water availability but not with temperature. PLoS ONE.

[57] Saatchi SS, Houghton RA, Dos Santos Alvala RC, Soares JV, Yu Y (2007). Distribution of aboveground live biomass in the Amazon basin. Glob Chang Biol.

[58] Vogel JG, Bond-Lamberty BP, Schuur EA, Gower ST, Mack MC, o'connell KE (2008). Carbon allocation in boreal black spruce forests across regions varying in soil temperature and precipitation. Glob Chang Biol.

[59] Zeng W, Duo H, Lei X, Chen X, Wang X, Pu Y (2017). Individual tree biomass equations and growth models sensitive to climate variables for Larix spp. in China. Eur J Forest Res.

[60] Tao S, Guo Q, Li C, Wang Z, Fang J (2016). Global patterns and determinants of forest canopy height. Ecology.

[61] Keith H, Mackey BG, Lindenmayer DB (2009). Re-evaluation of forest biomass carbon stocks and lessons from the world's most carbon-dense forests. PNAS.

[62] Lie Z, Xue L, Jacobs DF (2018). Allocation of forest biomass across broad precipitation gradients in China’s forests. Sci Rep.

