# Supplementary material for: Effects of climate and plant functional types on forest above-ground biomass accumulation
Source: Carbon Balance Manag. 2023 Mar 22;18:5. doi: 10.1186/s13021-023-00225-1 (PMC10035156; doi:10.1186/s13021-023-00225-1)
Supplement: Supplementary file 1 — Additional file 1. Data Collection. [file 13021_2023_225_MOESM1_ESM.docx]

**Effects of climate and plant functional types on forest above-ground biomass accumulation**

**Additional file 1. Data collection**

***AGB data (2.3 in the main text)***

Our database consisted of geolocation (longitude, latitude, and elevation), dominant tree species, and forest functional type, above-ground biomass (AGB) (Mg ha^-1^) (the measurements originally expressed in units of C were converted to units of dry organic matter using the IPCC default of *C* = 0*.*47 *×* AGB [63] and forest age. Specifically, the data were collected in two ways:

(1) Available databases:

1). The Global Forest Carbon database [33, 34]: ForC contains records of field-based measurements of forest carbon stocks, compiled from original publications and existing data compilations and databases. Measurement records included sites, dominant plant functional type, stand age, measurement methods and values. AGB data included in ForC are measured in the field directly or estimated by allometric relations. The database does not include modeled estimates or estimates based on remote sensing. The ForC authors removed sites from this data set if the sites had undergone significant management or other significant disturbances since the most recent stand initiation event. We selected 887 records from this data set that met our criteria, including 109 time series.

2). The Forest Biomass database of Eurasia [35]: this dataset was compiled from a combination of experiments undertaken by the authors and from scientific publications that recorded biomass, geographical location, tree species composition (the information of forest functional type was obtained on this basis), stand age, number of trees per hectare, number of trees selected for destructive sampling, etc. Data presented in this data set were collected using in-situ destructive sampling measurements. Specifically, a number of trees are selected, cut, and measured to assess the live biomass. We selected 4399 records from this data set that met our criteria, including 356 time series.

3). The Forest Biomass database of China [36]: this is a comprehensive forest biomass data set for China, from a wide range of survey data obtained from the literature, which was standardized and quality-checked. The data set included biomass data, geographical location, dominant tree species (the information of forest functional type was obtained on this basis), stand age, and biomass measurement methods. Approximately 50% of the AGB data was collected using destructive sampling measurements and approximately 50% of the AGB data was estimated by allometric equations. We selected 236 records from this data set that met our criteria, including 29 time series.

(2) Published literature

Additional targeted literature searches were conducted to identify further available data on the AGB analyzed here. Article searches and inclusion were performed following the guidelines suggested by PRISMA [64]. We systematically reviewed the literature (April 2021) with the Web of Science, using the following search terms: (above-ground biomass* OR AGB* OR carbon storage*) AND (forest* OR natural forest* plantation* OR planted forest*) AND (stand development* OR age*). The initial search included over 15 000 peer-reviewed studies, which were further screened to meet the following inclusion criteria: (a) natural forest or plantations with a developmental stage from young to mature to steady state; (b) above-ground biomass measurements with age sequences under discernible geolocation; (c) sites that had not undergone major natural disturbance with site mortality >10% of trees or thinning. We reviewed all the abstracts to identify accessible studies and to find studies that quantified carbon or biomass stocks. Next, the raw data were collected using the web address in the Data availability section provided by the author or extracted from tables and figures using WebSiteDigitizer-4 software. We drew 874 records from the literature that met our criteria, including 68 time series.

**Reference**

63. IPCC. Intergovernmental Panel on Climate Change (IPCC), An IPCC Special Report on the Impacts of Global Warming of 1.5 °C Above Pre-Industrial Levels and Related Global Greenhouse Gas Emission Pathways. 2018.

64. Liang XY, Zhang T, Lu XK, Ellsworth DS, BassiriRad H, You CM, et al. Global response patterns of plant photosynthesis to nitrogen addition: A meta-analysis. Glob Chang Biol. 2020; 26(6): 3585-600.
