# Supplementary material for: Effects of climate and plant functional types on forest above-ground biomass accumulation
Source: Carbon Balance Manag. 2023 Mar 22;18:5. doi: 10.1186/s13021-023-00225-1 (PMC10035156; doi:10.1186/s13021-023-00225-1)
Supplement: Supplementary file 2 — Additional file 2: Figure S1. A flowchart of the study. The blue arrow indicates the main working path, the red arrow indicates data input, and the yellow arrow indicates model evaluation. Figure S2. Age distributions of four forest functional types ((a) Broadleaf deciduous, (b) Broadleaf evergreen, (c) Needleleaf deciduous and (d) Needleleaf evergreen) at 0.5-degree grid from the Global Forest Age Dataset (GFAD) (Poulter et al., 2019). Figure S3. Residual fraction of the model prediction against the predicted AGB (Mg ha−1) across four forest types. The residual is defined as the difference between observed and predicted AGB. The residual fraction is the ratio between residual and predicted AGB. The red lines indicate the zero residual and the data points above it represent cases where predicted AGB is smaller than observed AGB. Figure S4. Cross-validation results of model fitting. For the data set of each forest type, we randomly chose 70% data points as the training set, to fit the model parameters and let the other data points be used as the testing set to evaluate the predictions. Figure S5. The comparison between predicted AGB (Mg ha−1) calculated based on the fitted model and a global wall-to-wall remote sensing-based AGB (Mg ha−1) maps made by Hu et al. (2016). The 1-km resolution wall-to-wall global forest AGB map was resampled and extracted at a 0.5-degree resolution. The mean residual is $33.32$33.32 Mg ha−1 and the mean residual fraction is 0.199. Figure S6. Global geographic distribution of the predicted current (2000–2010 era) forest AGB (Mg ha−1). The total AGB in each grid cell is calculated based on the age and proportion of the four forest functional types at a 0.5-degree resolution with the median value (a), [file 13021_2023_225_MOESM2_ESM.docx]

**Effects of climate and plant functional types on forest above-ground biomass accumulation**

**Additional file 2. Supplementary figures**

**
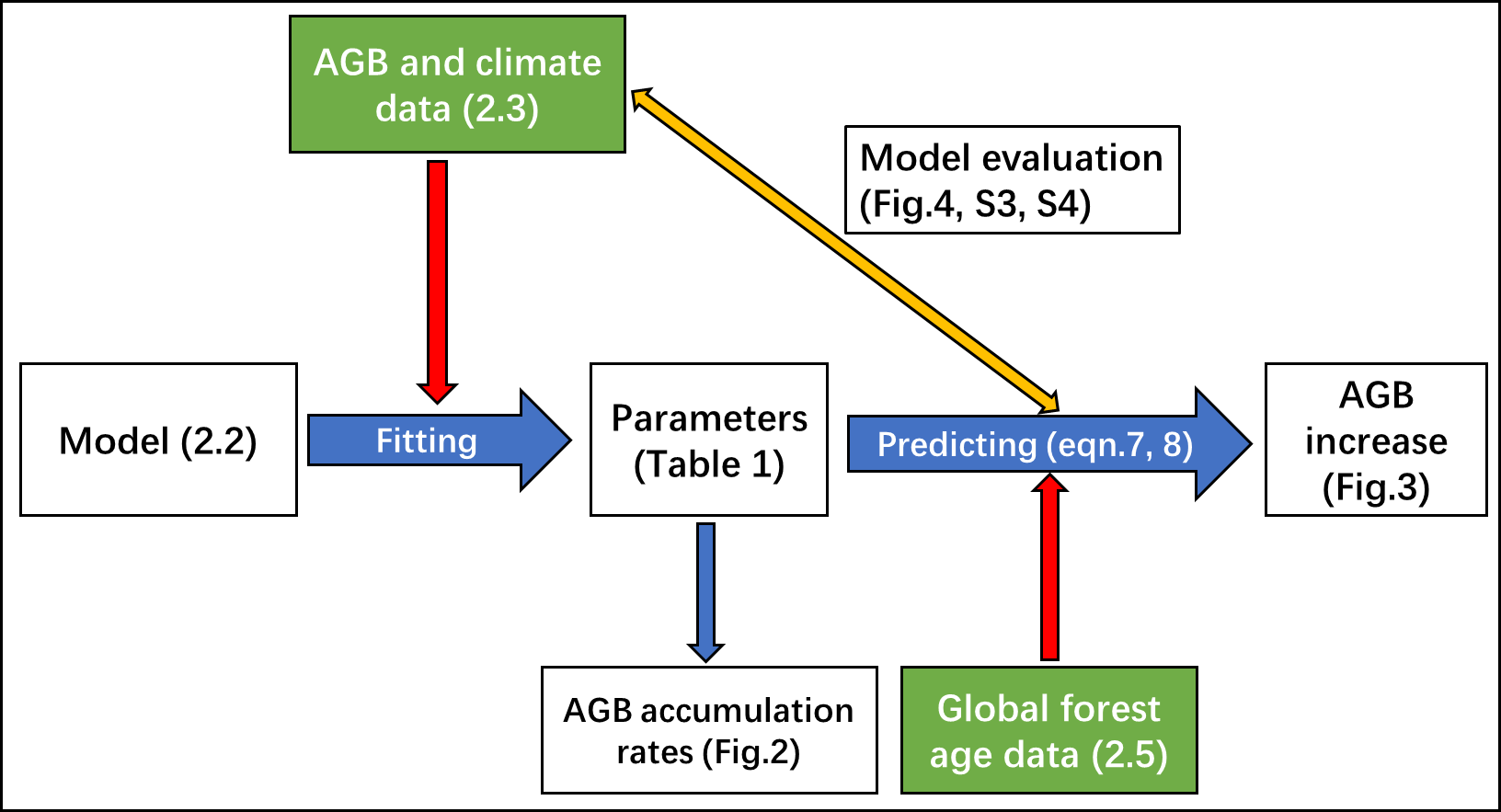
**

**Figure S1.** A flowchart of the study. The blue arrow indicates the main working path, the red arrow indicates data input, and the yellow arrow indicates model evaluation.


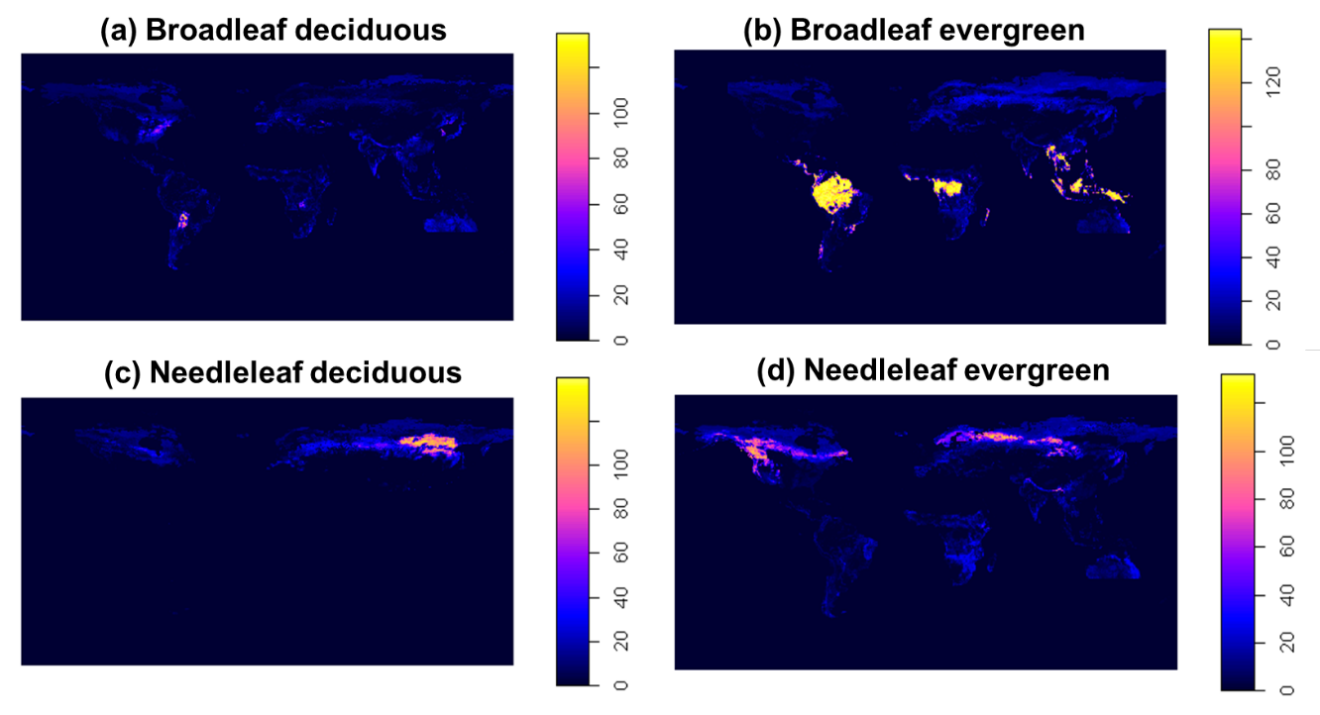


**Figure S2.** Age distributions of four forest functional types ((a) Broadleaf deciduous, (b) Broadleaf evergreen, (c) Needleleaf deciduous and (d) Needleleaf evergreen) at 0.5-degree grid from the Global Forest Age Dataset (GFAD) [41].


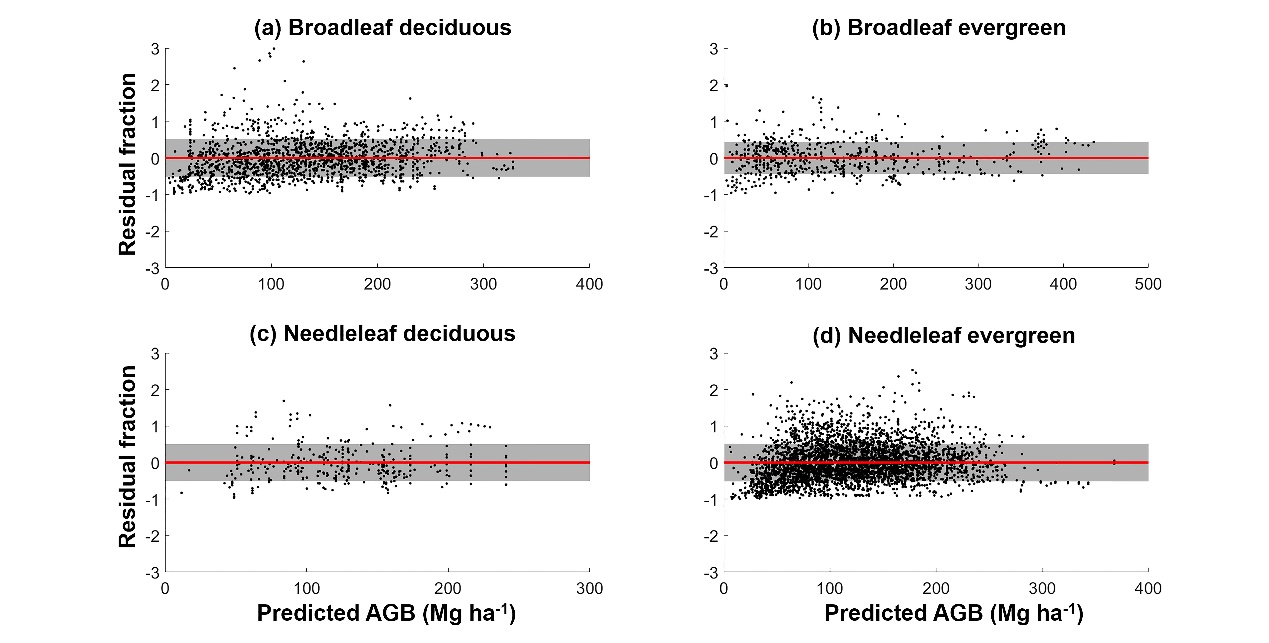


**Figure S3.** Residual fraction of the model prediction against the predicted AGB (Mg ha^-1^) across four forest types. The residual is defined as the difference between observed and predicted AGB. The residual fraction is the ratio between residual and predicted AGB. The red lines indicate the zero residual and the data points above it represent cases where predicted AGB is smaller than observed AGB.


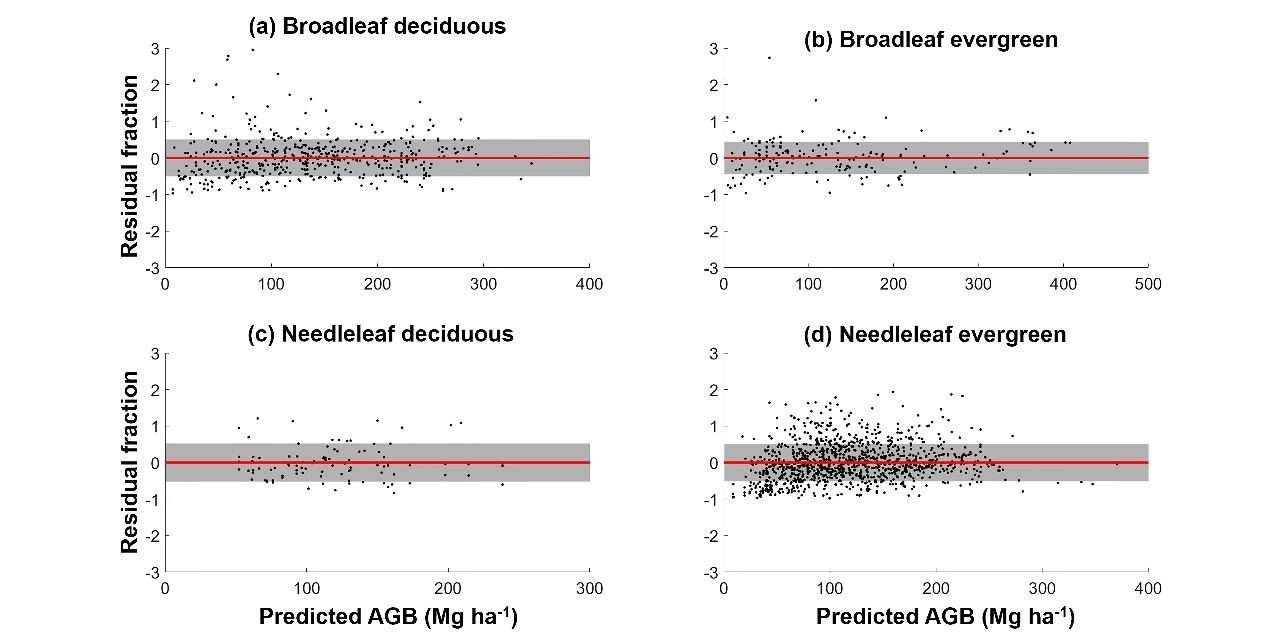


**Figure S4.** Cross-validation results of model fitting. For the data set of each forest type, we randomly chose 70% data points as the training set, to fit the model parameters and let the other data points be used as the testing set to evaluate the predictions.


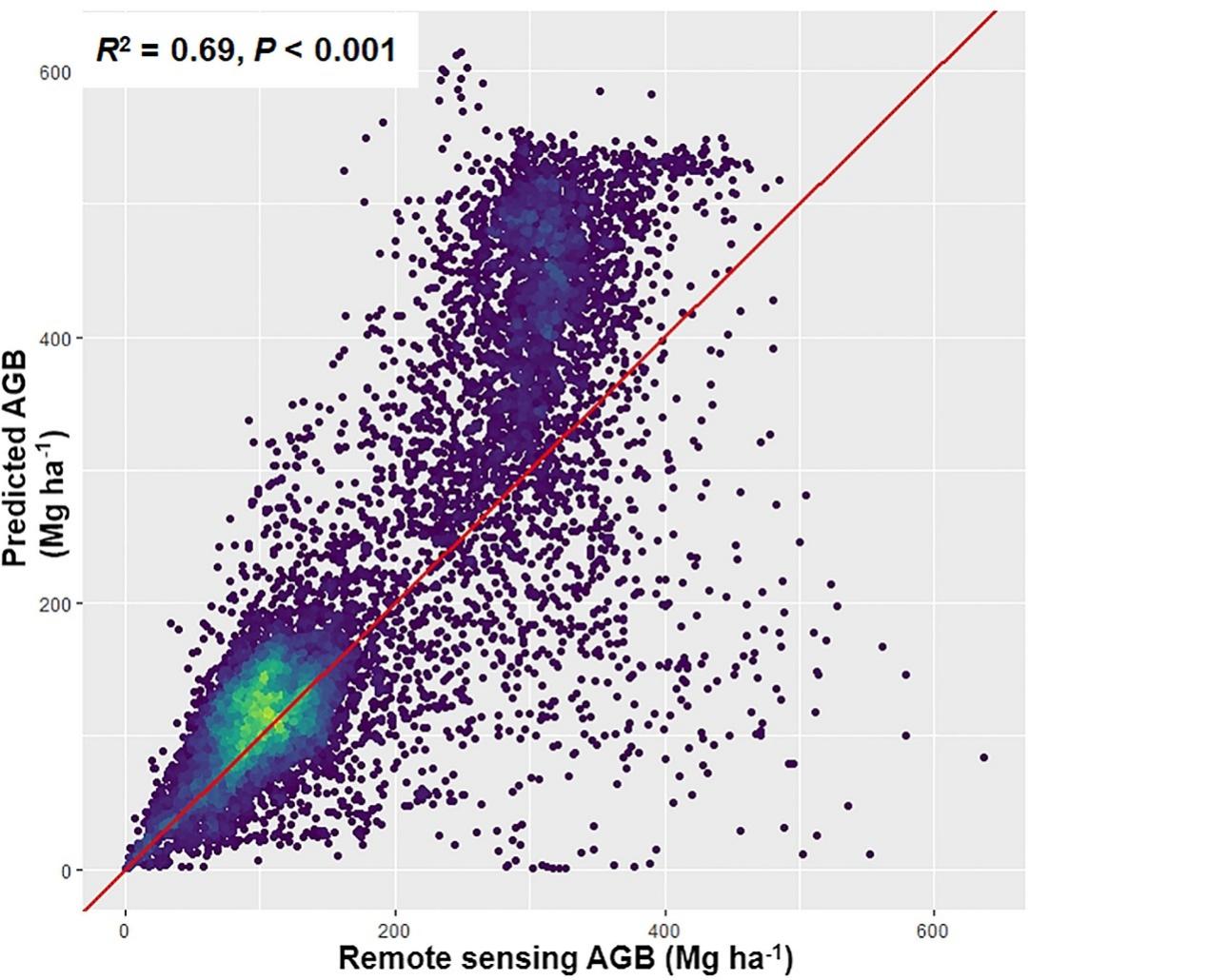


**Figure S5.** The comparison between predicted AGB (Mg ha^-1^) calculated based on the fitted model and a global wall-to-wall remote sensing-based AGB (Mg ha^-1^) maps made by Hu et al. (2016). The 1-km resolution wall-to-wall global forest AGB map was resampled and extracted at a 0.5-degree resolution. The mean residual is 33.32 Mg ha^-1^ and the mean residual fraction is 0.199.


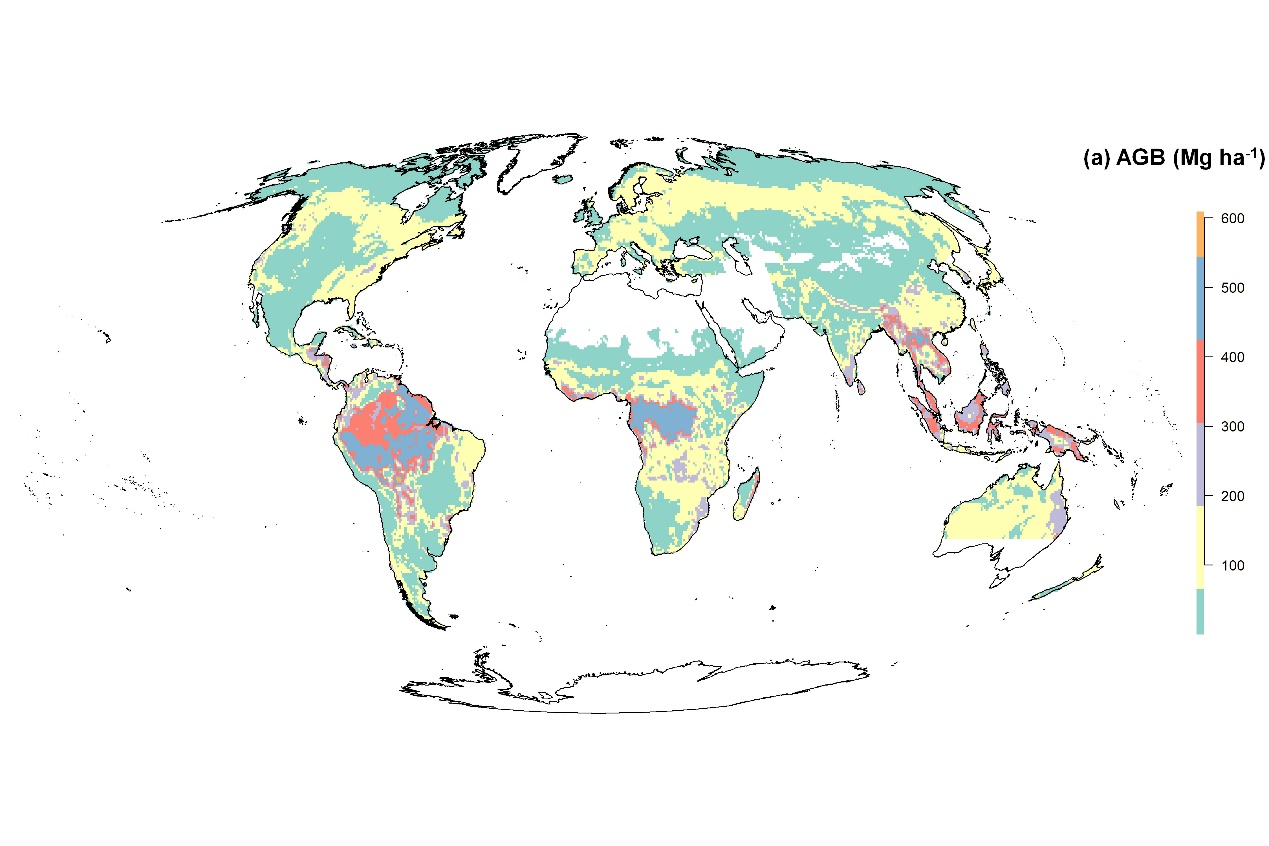


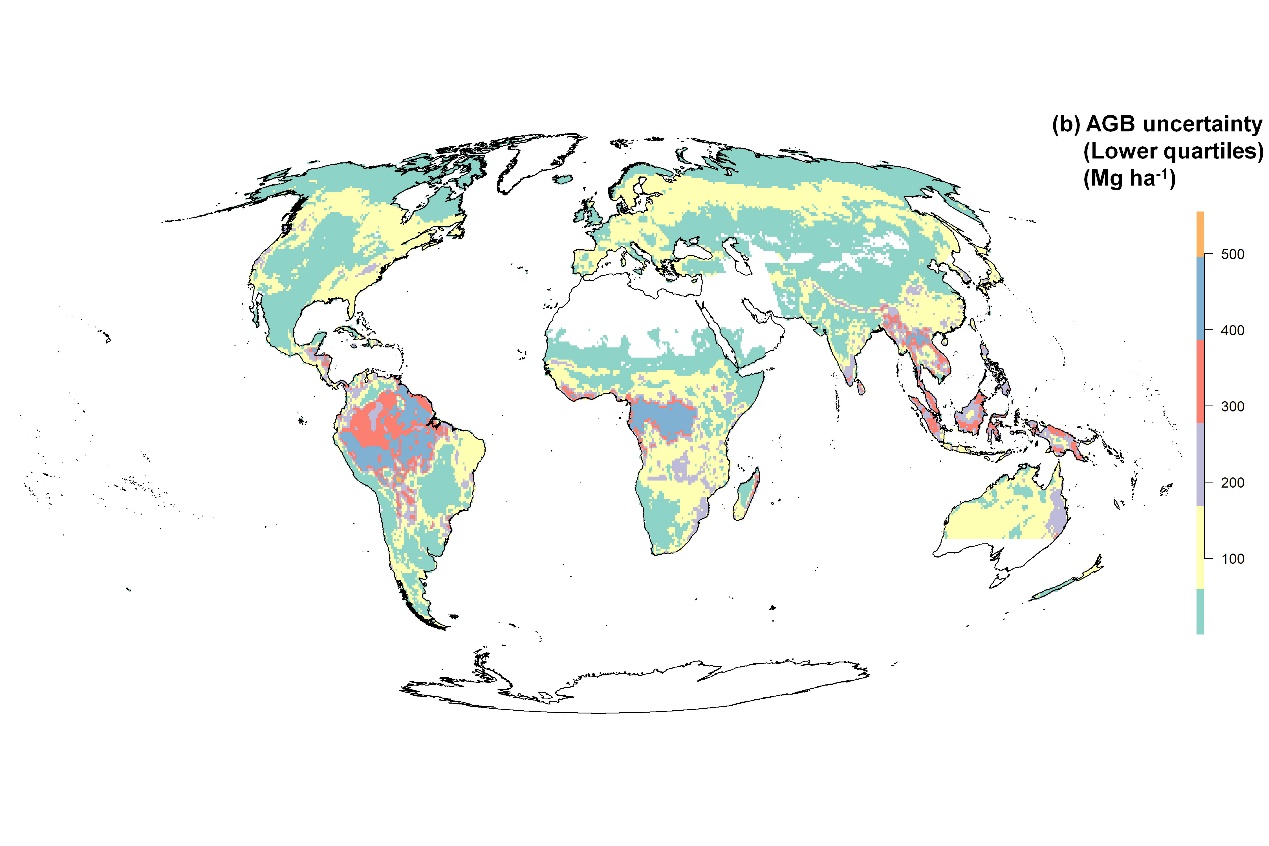


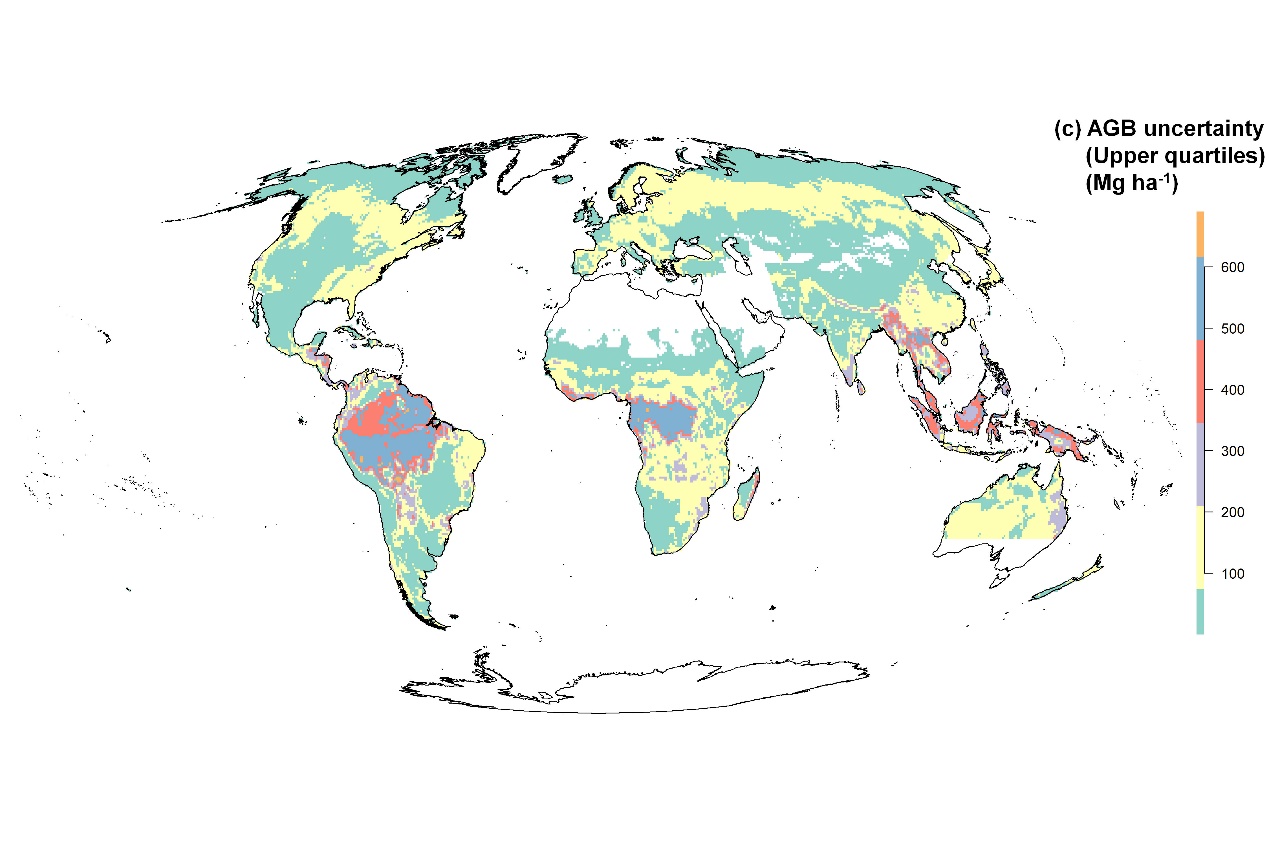


**Figure S6.** Global geographic distribution of the predicted current (2000–2010 era) forest AGB (Mg ha^-1^). The total AGB in each grid cell is calculated based on the age and proportion of the four forest functional types at a 0.5-degree resolution with the median value (a), lower quartiles (b) and upper quartiles (c) of the posterior distribution. The blank area in southern Australia occurs because no data for this area exist in the global forest age data set. Other blank areas show grid cells without any forests due to glaciers or extreme aridity.

**Table S1.** Sensitivity analysis of climate conditions on parameter estimation.

| **Forest type** | **Climate** | **Saturated above-ground biomass (*μ*)** | | **Initial biomass accumulation rate (*r*)** | |
| --- | --- | --- | --- | --- | --- |
|  |  | **MAP effect**  **(Mg ha^–1^ mm^–1^)** | **MAT effect**  **(Mg ha^–1^ °C^–1^)** | **MAP effect**  **(Mg ha^–1^ year^-1^**  **mm^–1^)** | **MAT effect**  **(Mg ha^–1^ year^-1^**  **°C^–1^)** |
| **Broadleaf deciduous** | 1970–2000  **1961–1969**  **2001–2009** | -0.2122  (-0.2693, -0.1418)  **-0.1635**  **(-0.2223, -0.0845)**  **-0.1057**  **(-0.1895, 0.0271)** | 19.0471  (15.3600, 22.6571)  **16.1286**  **(12.5129, 19.3986)**  **14.4243**  **(9.7400, 18.1071)** | 0.0071  (0.0062, 0.0079)  **0.0070**  **(0.0059, 0.0080)**  **0.0053**  **(0.0040, 0.0066)** | -0.0386  (-0.0729, -0.0043)  **-0.0029**  **(-0.0414, 0.0343)**  **0.0343**  **(-0.0143, 0.0786)** |
| **Broadleaf evergreen** | 1970–2000  **1961–1969**  **2000–2009** | -0.1905  (-0.2621, -0.1210)  **-0.1210**  **(-0.1708, -0.0671)**  **-0.1398**  **(-0.1949, -0.0845)** | 36.9300  (5.4271, 69.0714)  **42.6700**  **(11.9886, 62.3229)**  **25.9700**  **(-4.5600, 55.3829)** | -0.0032  (-0.0037, -0.0027)  **-0.0026**  **(-0.0030, -0.0023)**  **-0.0027**  **(-0.0031, -0.0023)** | -0.1429  (-0.2871, 0.0114)  **-0.3543**  **(-0.4557, -0.2629)**  **-0.2257**  **(-0.3443, -0.1000)** |
| **Needleleaf deciduous** | 1970–2000  **1961–1969**  **2000–2009** | -0.1964  (-0.3613, -0.0263)  **-0.2201**  **(-0.3739, -0.0731)**  **-0.2409**  **(-0.3983, -0.0871)** | 8.2000  (-4.3529, 19.4900)  **6.7400**  **(-2.6571, 16.3371)**  **8.2900**  **(-1.7571, 17.7000)** | 0.0028  (-0.0015, 0.0080)  **0.0010**  **(-0.0014, 0.0040)**  **0.0008**  **(-0.0015, 0.0034)** | 0.2486  (-0.0243, 0.4443)  **0.2914**  **(0.2014, 0.3829)**  **0.2914**  **(0.2129, 0.3743)** |
| **Needleleaf evergreen** | 1970–2000  **1961–1969**  **2000–2009** | 0.0874  (0.0064, 0.1745)  **0.0967**  **(0.0182, 0.1819)**  **0.1337**  **(0.0497, 0.2237)** | 22.3700  (18.7143, 26.0643)  **20.8171**  **(17.2429, 24.5614)**  **20.8571**  **(17.6386, 24.2929)** | -0.0002  (-0.0005, 0.0001)  **-0.0002**  **(-0.0005, 0.0000)**  **-0.0003**  **(-0.0006, 0.0000)** | 0.2686  (0.2514, 0.2857)  **0.2643**  **(0.2471, 0.2814)**  **0.2743**  **(0.2571, 0.2900)** |
